# miR-625-3p is upregulated in CD8+ T cells during early immune reconstitution after allogeneic stem cell transplantation

**DOI:** 10.1371/journal.pone.0183828

**Published:** 2017-08-30

**Authors:** Kriti Verma, Nidhi Jyotsana, Ivonne Buenting, Susanne Luther, Angelika Pfanne, Thomas Thum, Arnold Ganser, Michael Heuser, Eva M. Weissinger, Lothar Hambach

**Affiliations:** 1 Dept. of Hematology, Hemostasis, Oncology and Stem Cell Transplantation, Hannover Medical School, Hannover, Germany; 2 Integrated Research and Treatment Center for Transplantation (IFB-Tx), Hannover, Germany; 3 Institute of Molecular and Translational Therapeutic Strategies, Hannover Medical School, Hannover, Germany; 4 REBIRTH Excellence Cluster, Hannover Medical School, Hannover, Germany; 5 National Heart and Lung Institute, Imperial College London, London, United Kingdom; Universitat des Saarlandes, GERMANY

## Abstract

Alloreactive CD8+ T-cells mediate the curative graft-versus-leukaemia effect, the anti-viral immunity and graft-versus-host-disease (GvHD) after allogeneic stem cell transplantation (SCT). Thus, immune reconstitution with CD8+ T-cells is critical for the outcome of patients after allogeneic SCT. Certain miRNAs such as miR-146a or miR-155 play an important role in the regulation of post-transplant immunity in mice. While some miRNAs e.g. miR-423 or miR-155 are regulated in plasma or full blood during acute GvHD also in man, the relevance and expression profile of miRNAs in T-cells after allogeneic SCT is unknown. miR-625-3p has recently been described to be overexpressed in colorectal malignancies where it promotes migration, invasion and apoptosis resistance. Since similar regulative functions in cancer and T-cells have been described for an increasing number of miRNAs, we assumed a role for the cancer-related miR-625-3p also in T-cells. Here, we studied miR-625-3p expression selectively in CD8+ T-cells both in vitro and during immune reconstitution after allogeneic SCT in man. T-cell receptor stimulation lead to miR-625-3p upregulation in human CD8+ T-cells in vitro. Maintenance of elevated miR-625-3p expression levels was dependent on ongoing T-cell proliferation and was abrogated by withdrawal of interleukin 2 or the mTOR inhibitor rapamycin. Finally, miR-625-3p expression was analyzed in human CD8+ T-cells purified from 137 peripheral blood samples longitudinally collected from 74 patients after allogeneic SCT. miR-625-3p expression was upregulated on day 25 and on day 45, i.e. during the early phase of CD8+ T-cell reconstitution after allogeneic SCT and subsequently declined with completion of CD8+ T-cell reconstitution until day 150. In conclusion, this study has shown for the first time that miR-625-3p is regulated in CD8+ T-cells during proliferation in vitro and during early immune reconstitution after allogeneic SCT in vivo. These results warrant further studies to identify the targets and function of miR-625-3p in CD8+ T-cells and to analyze its predictive value for an effective immune reconstitution.

## Introduction

Allogeneic stem cell transplantation (SCT) is a curative treatment for haematological malignancies. [1, 2] Donor derived alloreactive CD8+ T cells play an important role in the curative graft versus leukaemia (GvL) effect, the viral specific immunity and the detrimental graft versus host disease (GvHD) after allogeneic SCT[3–5]. Thus, immune reconstitution with CD8+ T cells is a critical parameter for the outcome of patients after allogeneic SCT. Several external factors like the transplanted T cell dose, the level of T cell depletion and immunosuppression influence T cell reconstitution after allogeneic SCT[6]. However, little is known about intrinsic cellular parameters regulating T cell reconstitution.

There is increasing evidence that miRNAs play an important role in the regulation of post-transplant immunity[7]. miRNAs are small (18-22bp) non-coding RNAs that regulate gene expression by repressing specific target genes at the post transcriptional level. Nevertheless, the exact physiological and pathophysiological relevance of most T cell associated miRNAs is unknown. On cellular level, 71 of 420 highly characterized miRNAs are differentially expressed upon human T cell activation in vitro[8]. miRNAs regulate multiple functions in T cells such as TCR signaling, proliferation, differentiation, cytokine secretion and apoptosis[9] E.g. miR-146a upregulation upon TCR stimulation increases the overall TCR signaling and, thereby, enhances cell activation and cell expansion[10] miR-155 targets SOCS1, Ship1 and many other mRNAs that participate in type 1 interferon (IFN) signaling and promotes CD8+ T cell proliferation and survival[11, 12] miR-17-92 targets the tumor suppressors Pten, ID2, ID3 and the anti-apoptotic bcl-2 and enhances the cell cycle progression of T cells[13]. These known cellular functions of miRNAs suggest that miRNAs may also play a role in T cell mediated effects after allogeneic SCT, e.g. GvHD. Prevention of GvHD in mice can be achieved by overexpression of miR-146a[14] or by inhibition of the miR-17-92 cluster[15] or miR-155[16]. In man, high expression of miR-423, miR-199a-3p, miR-93*, and miR-377 in plasma[17] and low expression of miR-146a-5p and miR-155in whole blood can predict acute GvHD[18]. Thus, certain miRNAs are also differentially regulated after allogeneic SCT in man. To the best of our knowledge, miRNAs have not been studied selectively in human T cells after allogeneic SCT.

Several miRNAs overexpressed in activated T cells also play a role in cancer [19]. E.g. miR146a inhibits EGFR and NF-kB signaling and reduces the invasion and metastatic potential in breast and pancreatic cancer[19]. miR-155 increases cell proliferation through the activation of the JAK2/STAT3, AKT, NF-kB and Wnt/β-catenin signaling pathways in various malignancies [11, 20]. The miR-17-92 cluster is overexpressed in many types of cancer, promotes cell proliferation and sustains cell survival via suppressing TGF-β signaling and inhibiting the tumor suppressor Pten[21] These data suggest that functions like proliferation and migration are regulated by the same miRNAs in activated T cells and cancer cells. Recently, abnormal expression of the less characterized miR-625-3p has been described in primary colorectal carcinoma (CRC) and in CRC cell lines. [22, 23] Moreover, miR-625-3p has been detected in the circulation of malignant pleural mesothelioma patients. Overexpression of miR-625-3p in CRC cell lines promotes migration and invasion[23]. However, the expression and relevance of miR-625-3p had not been studied in T cells. Due to the similarities in regulative functions in cancer and T-cells previously shown for an increasing number of miRNAs we assumed also a differential expression of miR-625-3p in lymphocytes.

Here, we studied for the first time the expression dynamics of miR-625-3p in human CD8+ T cells both in vitro and after allogeneic SCT in vivo.

## Materials and methods

### Patients and sample collection

The study population consisted of human subjects who underwent allogeneic SCT between 2005 and 2015 at Hannover Medical School, Germany and 10 healthy donors. Patients were treated according to SCT protocols approved by the Institutional Review Board of the Hannover Medical School. Patients and donors gave written informed consent in accordance with the declaration of Helsinki. Analysis was performed with approval of the Institutional Review Board of the Hannover Medical School (1453–2012 and 2934–2015). aGvHD was graded according to the Glucksberg Score [24]. Peripheral blood mononuclear cells (PBMCs) were harvested from whole blood samples collected from 74 patients after allogeneic SCT on days 25 (range 17–32), 45 (range 33–62), 90 (range 64–126) and 150 (range 138–189). PBMCs were isolated by ficoll gradient and frozen in liquid nitrogen after supplementation in 80% RPMI-1640, 10% fetal calf serum (FCS, Sigma-Aldrich, Missouri, USA) and 10% dimethyl sulfoxide (DMSO, Sigma-Aldrich).

### Sorting of CD8+ T cells and RNA isolation

PBMCs thawed and cultured overnight in IMDM (Lonza, Basel, Switzerland), supplemented with 10% human serum (HS, Sigma-Aldrich) were labelled with anti-CD8-Alexa Fluor700 (Clone: RPA-T8, BD Biosciences, New Jersey, USA), anti-CD3-PE-Cy7 (Clone: UCHT1, Biolegend, San Diego, USA), anti-CD4-FITC(Clone OKT4, Biolegend) and 7AAD (Beckman Coulter, California, USA) and sorted on FACS Aria™ II (BD Biosciences) for 7AAD-CD3+CD8+ live T cells. Post-sorting analysis of purified subsets revealed greater than 98% purity. Mean CD8+ T cell count/μl was calculated as: (%CD8+ T cells gated from lymphocytes during sorting x total lymphocyte count/μl)/100 at the time of blood collection (± 2 days). Sorted CD8+ were pelleted by centrifugation, re-suspended in Trizol and immediately stored at -80°C. Total RNA was extracted using the miRNeasy Micro Kit (Qiagen, Hilden, Germany) and stored at -80°C after RNA quantification using a NanoDrop ND-1000 Spectrophotometer (Thermo scientific, Waltham, MA, USA). The phenotypic analysis was performed on FlowJo version 7.6.5 (Treestar, Ashland, USA).

### cDNA preparation and real time quantification of miRNA expression

For miRNA-specific complementary DNA synthesis, 10 ng total RNA was reversely transcribed using the TaqMan MicroRNA Reverse Transcription kit (Applied Biosystems, Foster city, CA, USA) in combination with multiplexed reverse transcription primers of miR-625-3p (TaqMan, Applied Biosystems). Single assay real-time-PCR for specific miRNAs was performed using 10 ng total cDNA with ABsolute Blue qPCR Mix (Thermo Scientific) and specific miRNA TaqMan Probes. RNU48 and U6 snRNA were used as internal controls for normalization using the ratio relative quantity (RQ) target gene/RQ normalization gene. RQ values were generated by the ViiA7 (Applied Biosystems) Real Time PCR instrument, using the formula RQ = 10[(ct-b)/a] (where ct is the threshold cycle, a and b are slope and intersection respectively, generated from a relative standard curve plotted from a pool of randomly selected samples).

### Cell proliferation assay

T cell proliferation was measured by quantification of 5-bromo-2’deoxyuridine (Roche, Basel, Switzerland) incorporated in dividing cells using an anti-BrdU antibody. In brief, CD8+ T cells were isolated from frozen PBMCs after resting overnight in IMDM supplemented with 10% human serum (HS, Sigma-Aldrich) by negative selection using the MACS CD8+ T cell isolation kit (Miltenyi, Bergisch Gladbach, Germany). Isolated CD8+ T cells were plated at a concentration of 2 × 10^5^ cells/well and stimulated with 2% Leucoagglutinin PHA-L (1 μg/mL, Sigma-Aldrich) in a final volume of 200 μl/well of flat bottom 96-well microtiter plates. BrdU was added 24 hours prior to harvest for each time point. Incorporated BrdU was quantified by ELISA in accordance to the instructions of the manufacturer (Roche) with medium alone as control.

### CD8+ T cell activation

2 × 10^5^ CD8+ T cells were plated in 200 μl/well after resting overnight in 10% HS/IMDM and stimulated with 2% Leucoagglutinin PHA-L (Sigma-Aldrich). T cells were harvested after 6, 24, 72, 120 and 168h, washed 1x with phosphate-buffered saline (PBS), labelled with anti-CD8-PE-Cy7 (clone: RPA-T8, BD Biosciences), anti-CD3-AlexaFluor700 (clone: UCHT1), anti-CD69-Pacific Blue (clone: FN50), anti-CD25-PE (clone: PC61), anti-CD71-APC (clone: CY1G4) all from Biolegend and 7AAD (Beckman Coulter) or L/D NEAR IR (Alexa Fluor® 750; Life technologies, Carlsbad, USA) and analyzed on BD LSR II. CMV and HA-1 specific T cells were stimulated with HLA-A2 CD14+ monocytes loaded with the A2/CMV peptide NLVPMVATV or A2/ HA-1 peptide VLHDDLLEA (MBL International, Woburn, USA) respectively.

### Statistics

All statistical analysis was performed using Prism Version 5 (GraphPad Software Inc., California, USA). A p value < 0.05 was considered statistically significant.

## Results

### miR-625-3p is upregulated in proliferating CD8+ T cells after strong TCR stimulation

**Fig 1:**
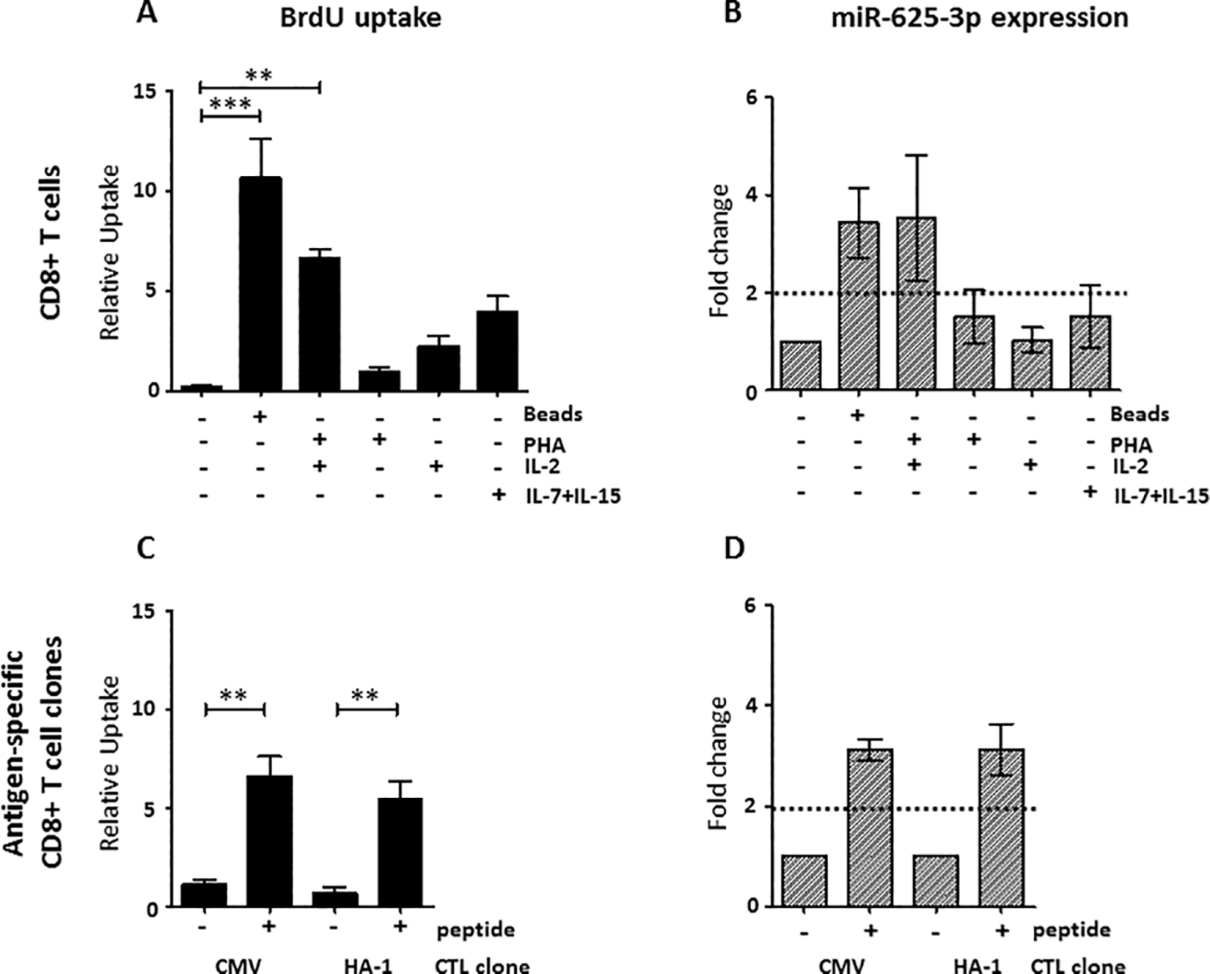
miR-625-3p upregulation in CD8+ T cells after stimulation. (A-B) CD8+ T cells were isolated from PBMCs of 3 healthy donors by MACS negative selection and stimulated with CD2/CD3/CD28 beads, PHA + IL-2, PHA alone, IL-2 or IL-7 + IL-15. BrdU uptake (A) and miR-625-3p expression (B) were measured 5 days after stimulation. (C-D) Established HLA-A2 restricted CMV and HA-1 specific CD8+ T-cell clones were stimulated with CD14+ monocytes loaded with CMV pp65 NLV or HA-1 peptide, respectively, in the presence of IL-2. BrdU uptake (C) and miR-625-3p expression (D) were measured 5 days after stimulation. Y-axis: (A, C) Relative uptake of BrdU was calculated as [absorbance of sample—absorbance of unstimulated control] / absorbance of unstimulated control; Statistical analysis for BrdU uptake was calculated by (A) one way ANOVA followed by Dunnett’s multiple comparison test for overall CD8+ T cells and (C) Unpaired t-test for antigen-specific cells. **indicates p<0.01, *** indicates p<0.001. (B, D) relative fold change in miR-625-3p expression was calculated by 2^-ΔΔct^ method using RNU48 and U6 snRNA as reference genes. Fold change above dotted line indicates significant fold change value >2. Data are representative of three independent experiments. All measurements were performed in duplicates.

miR-625-3p expression in total CD8+ T cells isolated from the peripheral blood of healthy donors was analyzed upon T cell proliferation induced by different stimuli, i.e. CD2/CD3/CD28 beads, the CD3-TCR binding lectin PHA [25, 26] and the cytokines IL-2 or IL-7+IL-15. Multiple testing demonstrated that only the difference in BrdU uptake between CD2/CD3/CD28 beads or PHA +IL-2 and the unstimulated condition was significant (p<0.01 and p = <0.001, one way ANOVA followed by Dunnett’s multiple comparison test, respectively) (**Fig 1A**). Accordingly, only stimulation with CD2/CD3/CD28 beads or with PHA +IL-2 resulted in a more than two fold miR-625-3p upregulation, while miR-625-3p expression remained low after stimulation with cytokines or PHA alone (**Fig 1B**). Thus, only conditions leading to strong T cell proliferation in our assay were associated with an increase of miR-625-3p expression. Of note, this increase of miR-625-3p expression was only present in proliferating CD8+ T cells stimulated via the CD3-TCR complex, i.e. via CD2/CD3/CD28 beads or PHA [25, 27] with IL-2 supplementation. Therefore, we analyzed whether also antigen-specific stimulation can upregulate miR-625-3p in CD8+ T cells. CD8+ T-cell clones specific for the human HLA-A2 restricted transplantation antigen HA-1 and for the CMV pp65 derived NLV peptide were stimulated with HA-1 or CMV peptide loaded APCs, respectively. Peptide stimulation resulted in a significant peptide dependent BrdU uptake (p = 0.003, p = 0.002, respectively, unpaired t-test, **Fig 1C**) and more than two fold upregulation of miR-625-3p (**Fig 1D**). Thus, CD8+ T cells proliferation induced by TCR stimulation along with co-stimulation provided by APCs was accompanied by miR-625-3p upregulation. Overall, these data show that strong TCR dependent stimuli either supported by cytokines or co-stimulation induce miR-625-3p upregulation in CD8+ T cells.

### miR-625-3p upregulation is a late event after T cell activation

**Fig 2:**
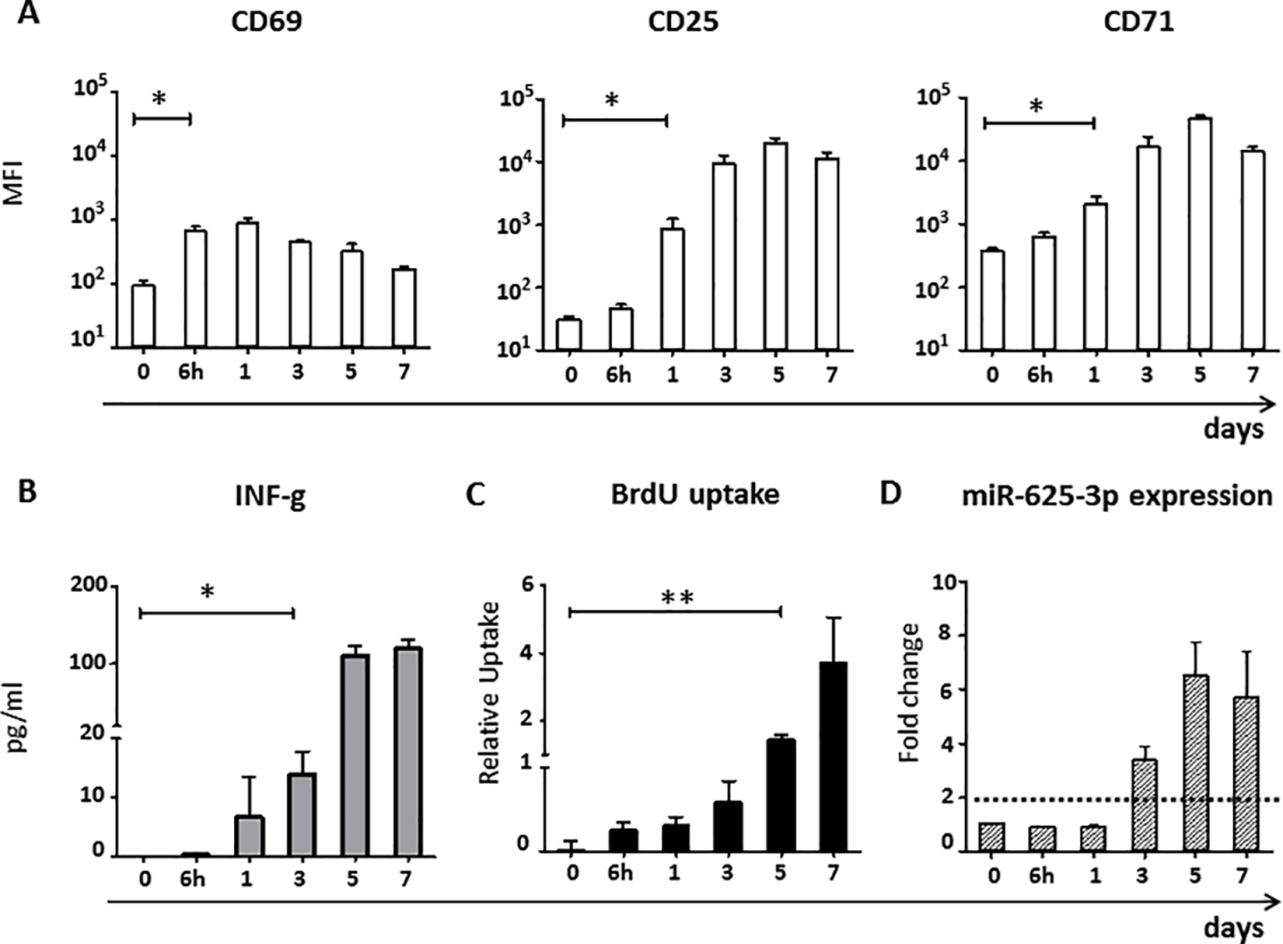
miR-625-3p upregulation is a late event after T cell activation. (A-D) CD8+ T cells isolated from PBMCs of 4 healthy donors were stimulated with PHA + 120 IU/ml IL-2 and (A) CD69, CD25 and CD79 surface expressions, (B) IFN-γ in the supernatant, (C) BrdU uptake and (D) intracellular miR-625-3p expression were measured after 0h, 6h, 1, 3, 5 and 7 days. Y-axis: (A) Mean fluorescence intensity (MFI), (B) Relative uptake of BrdU calculated as [absorbance of sample—absorbance of unstimulated control]/absorbance of unstimulated control, (C) IFN-γ levels in pg/ml, (D) Relative fold change in miR-625-3p expression calculated by 2^-ΔΔct^ method using RNU48 and U6 snRNA as reference genes. (A-C) Statistical analysis was calculated by paired t-test; *indicates p<0.05, **indicates p<0.01. (D) Fold change above dotted line indicates significant fold change value >2. Data are representative of three independent experiments. All measurements were performed in duplicates.

Subsequently, we studied the kinetics of miR-625-3p upregulation in parallel to T cell activation and proliferation. We stimulated CD8+ T cells with PHA, supplemented with IL-2 every two days and measured the surface expression of the activation markers CD69, CD25 and CD71; IFN-γ in the supernatant; BrdU uptake and intracellular miR-625-3p after 0h, 6h, 1, 3, 5 and 7 days after stimulation. T cells significantly upregulated the early T cell activation marker CD69 after 6h (p = 0.014) and the late activation markers CD25 and CD71 on day 1 (p = 0.018, p = -0.035, respectively, all paired t-test, **Fig 2A**), which is in accordance to previous reports[28]. Significant increase in IFN-γ levels (p = 0.037) and miR-625-3p expression (>2 fold change) was observed on day 3 preceding CD8+ T cell proliferation detected by a significant increase in BrdU uptake at day 5 post stimulation (p = 0.005, all paired t-test, **Fig 2B–2D**). Thus, miR-625-3p is detectable only late after upregulation of T cell activation surface markers and associates well with T cell proliferation and IFN-γ release.

### miR-625-3p upregulation in CD8+ T cells is transient and proliferation-dependent

**Fig 3:**
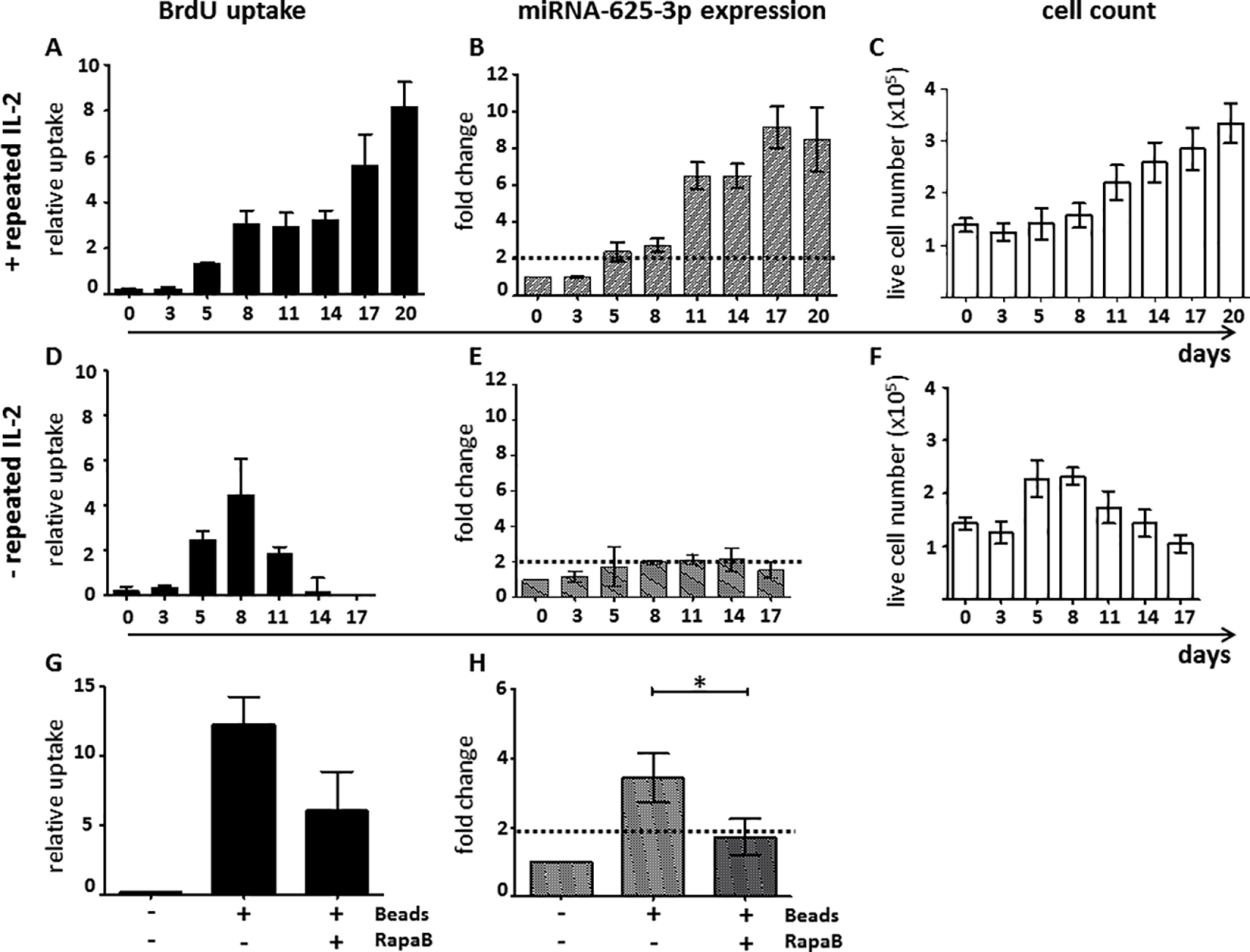
miR-625-3p expression in CD8+ T cells in relation to proliferation. (A-F) CD8+ T cells isolated from PBMCs of 4 healthy donors were stimulated with PHA and 80 IU/ml IL-2 was repeatedly added every second day (A-C) or only on day 2 (D-F). BrdU uptake (A, D), miR-625-3p expression (B, E) and viable cell counts determined by trypan blue staining (C, F) were measured on different days until day 20 (A-C) or 17 (D-F) after stimulation. (G, H) CD8+ T cells isolated from PBMCs of 3 healthy donors were stimulated with CD2/CD3/CD28 beads in the presence or absence of rapamycin (RapaB). BrdU uptake (G) and miR-625-3p expression (H) were measured on day 5 after stimulation. X axis: (A-F) days post SCT. Y-axis: (A,D,G) Relative uptake of BrdU was calculated as [absorbance of sample—absorbance of unstimulated control] / absorbance of unstimulated control. (B,E,H) Relative fold change in miR-625-3p expression was calculated by 2^-ΔΔct^ method using RNU48 and U6 snRNA as reference genes. All measurements were performed in duplicates. Fold change above dotted line indicates significant fold change value >2. Statistical comparisons were performed by paired t-test; *indicates p<0.05.

So far, our data suggest that miR-625-3p expression is linked to the initiation of T cell proliferation. In order to investigate the dependency of miR-625-3p expression on ongoing T cell proliferation, we studied the long-term kinetics of BrdU uptake, miR-625-3p expression and live cell counts in CD8+ T cells after PHA + IL-2 stimulation with (**Fig 3A–3C**) or without (**Fig 3D–3F**) repeated IL-2 supplementation until day 20 or 17, respectively. Samples with repetitive IL-2 supplementation showed a persistent increase of BrdU uptake (**Fig 3A**), miR-625-3p (**Fig 3B**) and cell numbers (**Fig 3C**). In contrast, samples without repetitive addition of IL-2 showed only a transient BrdU uptake (**Fig 3D**) and increase of cell numbers (**Fig 3F)** but no significant miR-625-3p expression (**Fig 3E**). Thus, miR-625-3p is strongly dependent on the continuous provision of a proliferative stimulus. Subsequently, we investigated whether inhibition of proliferation of CD2/CD3/CD28 bead stimulated CD8+ T cells using the mTOR inhibitor rapamycin also inhibits miR-625-3p expression (**Fig 3G and 3H**). While BrdU uptake was considerably inhibited (**Fig 3G**), miR-625-3p expression was almost abrogated by rapamycin (**Fig 3H**). Similar results were found for antigen-specific T cells (**S1 Fig**). Overall, these data suggest that although TCR stimulation upregulates miR-625-3p expression, maintenance of elevated miR-625-3p expression is dependent on the ongoing T cell proliferation.

### miR-625-3p is not regulated in peripheral blood CD8+ T cells during acute GvHD

**Fig 4:**
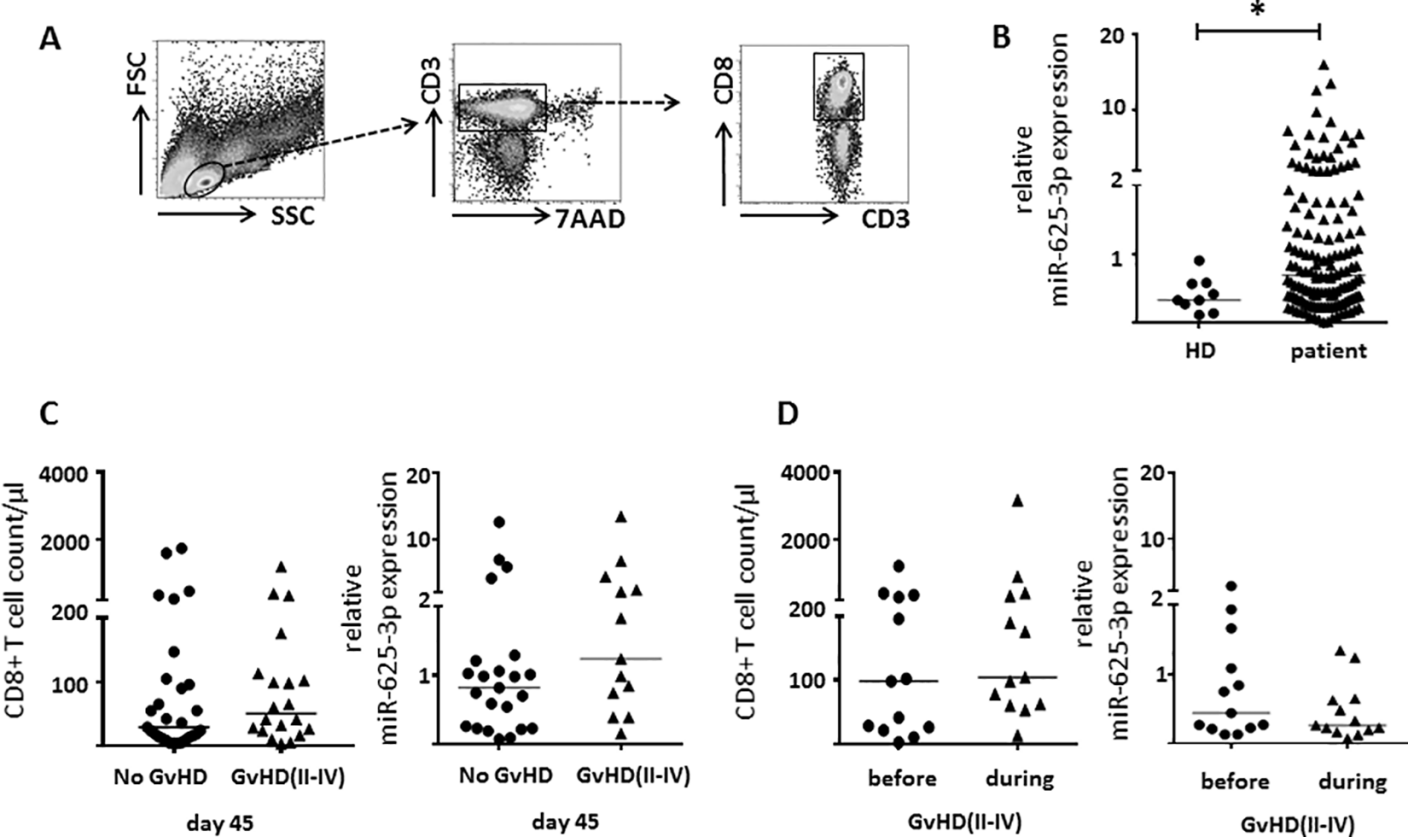
miR-625-3p in CD8+ T cells in patients with GvHD. (A) Viable CD3+CD8+ T cells were isolated by FACS sorting from peripheral blood samples and miR-625-3p expression was determined. (B) Relative miR-625-3p expression in CD8+ T cells isolated from 137 peripheral blood samples of 74 patients collected on days 25, 45, 90 and 150 after allogeneic SCT (triangles) compared to healthy donors (n = 9, circles). (C) Mean CD8+ T cell count / μl at the time of blood collection (left) and relative miR-625-3p expression in CD8+ T cells (right) isolated from peripheral blood samples collected on day 45 (±5 days) after allogenic SCT from patients without (n = 20, circles) and with (n = 13, triangles) severe GvHD (grade II-IV). (D) Mean CD8+ T cell count / μl at the time of blood collection (left) and relative miR-625-3p expression in CD8+ T cells (right) of 13 patients with severe GvHD (grade II-IV) determined in the last sample before GvHD onset (circles) and in a sample during GvHD (triangles). Relative miR-625-3p expression in individual patient samples calculated by miR-625-3p [RQ]/mean RNU48/U6 snRNA [RQ]. RQ: relative quantity. Median is shown as a bar. All measurements were performed in duplicates. Statistical comparisons between independent samples were performed by Mann-Whitney U test (B-C) and between dependent samples by Wilcoxon matched-pairs signed rank test (D). * indicates p<0.05.

So far, our in vitro data indicate a close association between miR-625-3p expression and T cell proliferation in vitro. This lead to the hypothesis that miR-625-3p might also be upregulated in CD8+ T cells during strong immune responses such as severe acute GvHD grade II-IV after allogeneic SCT. In order to identify a potential association between miR-625-3p and severe acute GvHD, miR-625-3p expression was analyzed in peripheral blood CD8+ T cells isolated from 137 longitudinally collected peripheral blood samples of 74 patients after allogeneic SCT (**Table 1**). CD8+ T cells were isolated by FACS sorting from peripheral blood samples collected according to the sorting strategy depicted in **Fig 4A**. Overall, miR-625-3p expression in CD8+ T cells was significantly higher (p = 0.031, Mann-Whitney U test) with high variability in peripheral blood of patients after allogeneic SCT compared to healthy donors (**Fig 4B**). In our cohort, 43% (32/74) of the patients developed acute GvHD II-IV with a median onset on day 47 (range 18–158) after allogeneic SCT. Therefore, peripheral blood samples collected on day 45 after allogeneic SCT were used to analyze in all patients whether miR-625-3p expression in CD8+ T cells was dysregulated in patients with acute GvHD grade II-IV within the observation period. CD8+ T cell reconstitution on day 45 did not significantly differ between patients with and without acute GvHD (p = 0.355, Mann-Whitney U test, **Fig 4C**). Moreover, there were no statistically significant differences in miR-625-3p expression between CD8 T cells derived from patients with or without acute GvHD (p = 0.177, Mann-Whitney U test, **Fig 4C**). Subsequently, we analyzed whether miR-625-3p expression in CD8+ T cells might predict the acute GvHD risk in patients with acute GvHD grade II-IV by comparing the last samples collected before onset of acute GvHD with samples collected during acute GvHD. Again, CD8 T cell counts did not significantly differ between samples before and during acute GvHD (p = 0.273, Wilcoxon matched-pairs signed rank test, **Fig 4D**). Moreover, miR-625-3p expression in CD8+ T cells did not significantly differ between samples collected before and during severe acute GvHD (p = 0.168, Wilcoxon matched-pairs signed rank test, **Fig 4D**). In conclusion, miR-625-3p expression in CD8+ T cells was not a biomarker of severe acute GvHD in our cohort.

**Table 1:** Patient characteristics.

**Factor**	**Characteristic**	**Patient Cohort n = 74, N (%)**
**Gender**	**Male**	45 (61)
	**Female**	29 (39)
**Median age**		52
**Diagnosis**	**AML / MDS**	43 (58)
	**ALL**	6 (8)
	**CML**	2 (3)
	**AA**	3 (4)
	**NHL / CLL**	7 (9)
	**Others**	4 (5)
**Donor**	**MRD**	23 (31)
	**MUD**	42 (57)
	**MMUD**	9 (12)
**Graft**	**PBSC**	68 (92)
	**BM**	5 (7)
	**BM+PBPC**	1 (1)
**Conditioning**	**Myeloablative**	24 (32)
	**RIC**	50 (68)
**GvHD Prophylaxis**	**CSA/MMF**	53 (72)
	**CSA/MTX**	21 (28)

**Abbreviations:** SD, standard deviation; AML, acute myeloid leukaemia; MDS, myelodysplastic syndrome; ALL, acute lymphoblastic leukaemia; CML, chronic myeloid leukaemia; AA, aplastic anaemia; NHL, Non-Hodgkin’s lymphoma; CLL, chronic lymphoid leukaemia; MRD, HLA matched related donor; MUD, HLA matched unrelated donor; MMUD, HLA mismatched unrelated donor; PBSC, peripheral blood stem cells; BM, bone marrow; RIC, reduced intensity conditioning; CSA, Cyclosporin A; MMF, mycophenolate mofetil; MTX, methotrexate.

### miR-625-3p is upregulated in CD8+ T cells during immune reconstitution after allogeneic SCT

**Fig 5:**
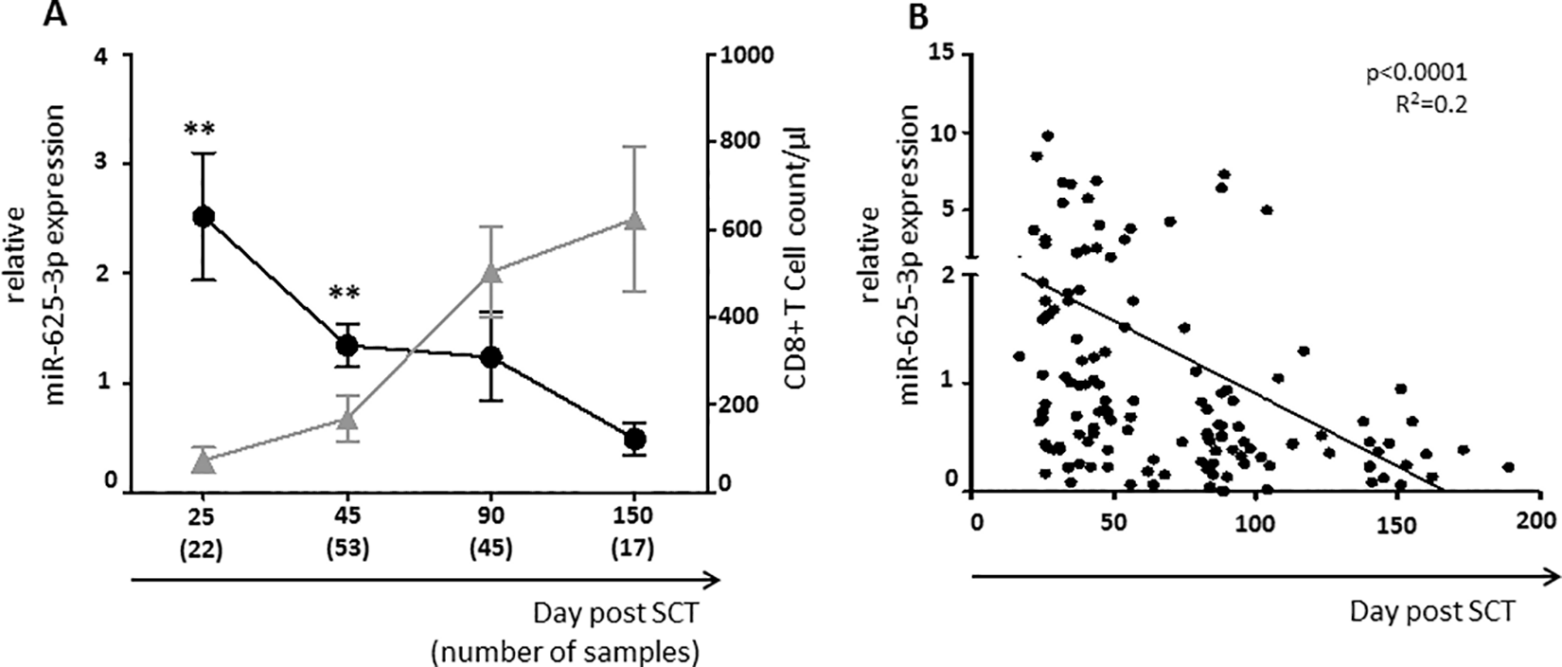
miR-625-3p expression is regulated during CD8+ T cell reconstitution in vivo. (A) Viable CD8+ T cells were quantified (grey triangles) and isolated by FACS sorting from peripheral blood samples collected on days 25 (n = 22), 45 (n = 53), 90 (n = 45) and 150 (n = 17) after allogeneic SCT. Subsequently, the relative miR-625-3p expression (black circles) was determined. Left Y-axis: Mean relative miR-625-3p expression calculated by miR-625-3p [RQ]/mean RNU48/U6 snRNA [RQ]. RQ: relative quantity. Right Y-axis: Mean CD8+ T cell count /μl at the time of blood collection (±5 days). Error bars indicate standard error of mean. All measurements were performed in duplicates. Statistical comparisons was calculated in comparison with the miR-625-3p expression in CD8+ T cells of healthy donors by Mann-Whitney U test; *indicates p<0.05. (B) Correlation analysis between relative miR-625-3p expression in patient derived CD8+ T cells (black circles) and the exact time of collection after allogeneic SCT is shown. The relationship between collection time and miR-625-3p expression in CD8+ T cells was analyzed by Spearman correlation coefficient (R^2^) analysis.

Rapid CD8+ T cell expansion in the peripheral blood is a hallmark of early immune reconstitution after allogeneic SCT. Therefore, we quantified miR-625-3p levels in CD8+ T cells on days 25, 45, 90 and 150 after allogeneic SCT in relation to CD8 T cell counts in the peripheral blood at the respective time points. In accordance with previous reports [29], CD8+ T cell counts were rapidly increasing from day 25 to day 90 after allogeneic SCT, while they hardly differed between day 90 and day 150 suggesting that CD8+ T cell reconstitution was largely completed by day 90 in our cohort (**Fig 5A**). On day 25 and on day 45, i.e. during rapid CD8+ T cell reconstitution after allogeneic SCT, miR-625-3p expression in CD8+ T cells was significantly higher than in healthy individuals who are characterized by a homeostasis in T cell numbers (p = 0.0015, p = 0.0077, respectively, Mann-Whitney U test). In contrast, on day 90 and on day 150, i.e. when CD8+ T cell reconstitution was largely completed, miR-625-3p expression in CD8+ T cells was comparable to healthy donors (**Fig 5A**). These data suggest that miR-625-3p expression in CD8+ T cells is differentially regulated during distinct phases of CD8+ T cell reconstitution after allogeneic SCT. Subsequently, we studied miR-625-3p expression in CD8+ T cells in relation to the sampling time point post-transplant. miR-625-3p levels in CD8+ T cells were significantly and negatively correlated with the time of sample collection after allogeneic SCT (p<0.0001, Spearman R^2^ = 0.2) (**Fig 5B**). Thus, high miR-625-3p expression in CD8+ T cells was associated with the rapid increase of CD8+ T cell numbers early after allogeneic SCT and declined with the time post-transplant.

## Discussion

In this study, we show for the first time the association of the cancer-related miR-625-3p with human CD8+ T cell activation and proliferation in vitro. To the best of our knowledge, the presented analysis is the first study on miRNA expression in isolated human CD8+ T cells after allogeneic SCT and shows that miR-625-3p is highly regulated during CD8 T cell reconstitution. In our in vitro experiments, miR-625-3p expression in human CD8+ T cells was induced by strong proliferative stimuli that involved triggering of the CD3-TCR complex, i.e. via CD2/CD3/CD28 beads, PHA with IL-2 supplementation or antigen-specific stimulation using peptide loaded APCs. PHA or IL-2 alone or the homeostatic cytokines IL-7 and IL-15 were capable to induce T cell proliferation to a lower extent as known from previous studies[30] but insufficient to induce miR-625-3p expression. In order to ascertain whether TCR dependency is mandatory for miR-625-3p upregulation, further studies on miR-625-3p expression in human T cells stimulated via TCR independent mechanisms are required such as CD31 triggering or transfection of P2X receptors regulating autocrine T cell activation.[31, 32] Our studies on miR-625-3p expression in relation to other cellular functions after TCR dependent T cell stimulation revealed that miR-625-3p is upregulated only after first signs of T cell activation, including CD69 (6h) or CD25 and CD71 (1 day) expression on the cell surface, IFN-γ release (3d) and largely parallels the kinetics T cell proliferation. Thus, miR-625-3p upregulation is a late marker of T cell activation. The strong association of miR-625-3p expression with high degrees of CD8+ T cell proliferation was further supported by the necessity of repetitive IL-2 supplementation to maintain high miR-625-3p levels and the lack of significant miR-625-3p upregulation upon withdrawal of IL-2. Finally, the mTOR inhibitor rapamycin which inhibits the T cell proliferation by negatively regulating cell cycle proteins and cytokine signaling mechanisms [33–35] lead to significant inhibition of miR-625-3p upregulation. This suggests that miR-625-3p might play a role downstream of the TCR signaling cascade. However, it remains unclear whether miR-625-3p directly regulates mTOR pathway or has a functional effect on CD8+ T cell proliferation via other pathways that may provide a survival advantage, e.g. apoptosis resistance, cytokine secretion or enhanced TCR signaling. Recently, overexpression of miR-625-3p in colorectal cancer cell lines had been shown to provide resistance to apoptosis via downregulating p38-MAPK which is involved in phosphorylation and activation of the pro-apoptotic bcl-2 gene[22]. Transfection experiments with miR-625-3p overexpression and silencing systems for e.g. using anti-sense miRNA targeting miR-625-3p are needed to refine the target molecules of miR-625-3p and to elucidate the function of miR-625-3p in human CD8+ T cells.

Due to the observed close association between miR-625-3p expression and T cell proliferation in vitro, we assumed that miR-625-3p might also be upregulated in CD8+ T cells during strong immune responses such as severe acute GvHD in vivo. Broadly expressed minor histocompatibility antigens (mHag) e.g. H-Y have been identified as the in situ targets of CD8+ T cells mediating GvHD.[36] However, only few GvHD associated mHags have been molecularly characterized to date.[37, 38] Therefore, we performed our analysis of miR-625-3p in the overall CD8+ T cell population assuming that these may comprise sufficient mHag specific T cells to allow detection of GvHD related miR-625-3p regulation. However, there was no difference in miR-625-3p expression in CD8+ T cells on day 45 after allogeneic SCT in patients with and without severe acute GvHD. Moreover, miR-625-3p expression in CD8+ T cells was not predictive for severe acute GvHD in patients with acute GvHD. Similar to previous reports[39], the absolute CD8 T cell counts did not significantly increase during acute GvHD in our cohort. Thus, the overall CD8+ T cell compartment might not have proliferated sufficiently during GvHD to allow detection of miR-625-3p upregulation. Additionally, mHag specific T cells within the CD8+ T cell compartment expanding during GvHD[40] might have been present in too small numbers to influence miR-625-3p expression.

Assuming that proliferation in the overall CD8+ T cell compartment is too low during acute GvHD to detect miR-625-3p upregulation in vivo, we investigated miR-625-3p expression during CD8+ T cell reconstitution after allogeneic SCT. Indeed, miR-625-3p expression in CD8+ T cells was highly upregulated shortly after allogeneic SCT when peripheral T cell numbers were still low and declined down to levels comparable to healthy donors in parallel with reaching maximum CD8+ T cell numbers on day 150 post-transplant. These data are in accordance with our in vitro data showing a close association between the levels of CD8+ T cell proliferation and of miR-625-3p expression. Thus, high miR-625-3p expression might indicate an effective CD8+ T cell reconstitution after allogeneic SCT. Additionally, miR-625-3p expression might provide insights into the mechanisms during early CD8+ T cell reconstitution. Namely, our in vitro data indicate the importance of TCR stimulation in comparison to only cytokines for the upregulation of miR-625-3p in CD8+ T cells. Thus, fast T cell proliferation in the lymphopenic host following allogeneic SCT might not only be driven by homeostatic cytokines[41] but also–as suggested earlier [42]–by TCR interactions with e.g. self MHC/peptides.

In conclusion, this study has demonstrated for the first time that the expression of cancer-related miR-625-3p is upregulated in CD8+ T cells in vitro upon TCR mediated activation and is tightly linked to the level of CD8+ T cell proliferation. High expression of miR-625-3p at an early time point during CD8+ T cell expansion after allogeneic SCT suggests that miR-625-3p might be applicable as intracellular biomarker for an effective immune reconstitution. Additional studies are required to identify the targets and function of miR-625-3p in human CD8+ T cells and to confirm the predictive value of miR-625-3p for the speed of CD8+ T cell reconstitution.

## Supporting information

S1 Fig Inhibition of proliferation in CMV and HA-1 specific CTL clones decreases miR-625-3p upregulation. HLA-A2 restricted CMV and HA-1 specific CD8+ CTL clones were stimulated by CD2/CD3/CD28 beads in the presence or absence of Rapamycin B and kept in culture for 4 days. Y axis: (A) Relative uptake of BrdU was calculated as [absorbance of sample—absorbance of unstimulated control] / absorbance of unstimulated control. (B) Relative fold change in miR-625-3p expression was calculated by 2^-ΔΔct^ method using RNU48 and U6 snRNA as reference genes.
(TIF)
