# Supplementary figures and images for: miR-625-3p is upregulated in CD8+ T cells during early immune reconstitution after allogeneic stem cell transplantation
Source: PLoS One. 2017 Aug 30;12(8):e0183828. doi: 10.1371/journal.pone.0183828 (PMC5576678; doi:10.1371/journal.pone.0183828)

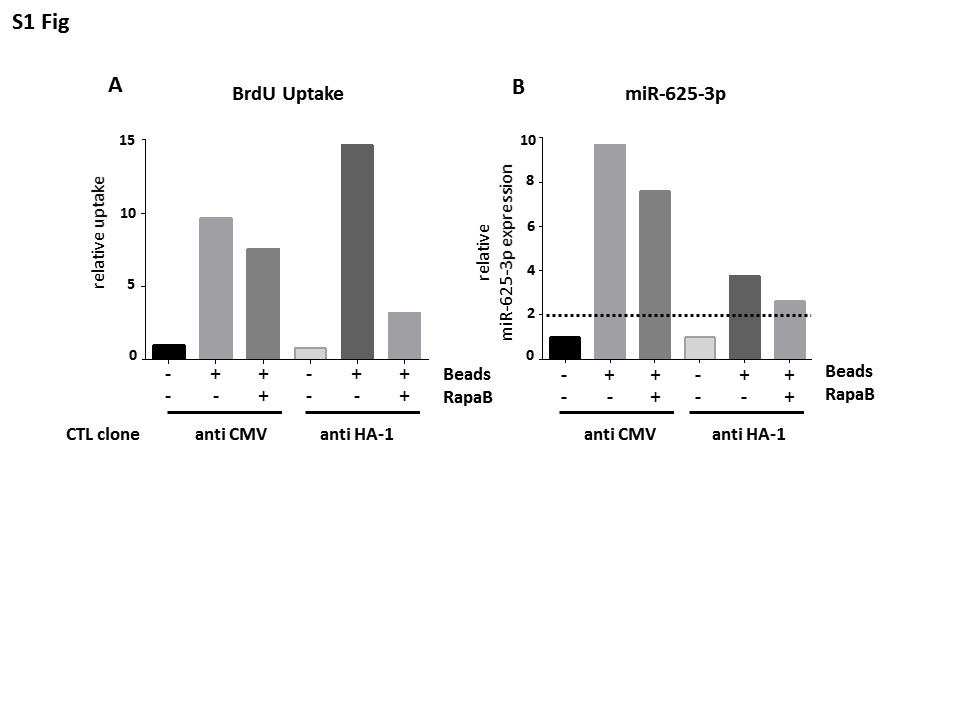

Supplement: S1 Fig — HLA-A2 restricted CMV and HA-1 specific CD8+ CTL clones were stimulated by CD2/CD3/CD28 beads in the presence or absence of Rapamycin B and kept in culture for 4 days. Y axis: (A) Relative uptake of BrdU was calculated as [absorbance of sample—absorbance of unstimulated control] / absorbance of unstimulated control. (B) Relative fold change in miR-625-3p expression was calculated by 2-ΔΔct method using RNU48 and U6 snRNA as reference genes. (TIF) [file pone.0183828.s001.tif]
